# The role of macrophages in gastric cancer

**DOI:** 10.3389/fimmu.2023.1282176

**Published:** 2023-12-06

**Authors:** Jiaqing Zhang, Can Hu, Ruolan Zhang, Jingli Xu, Yanqiang Zhang, Li Yuan, Shengjie Zhang, Siwei Pan, Mengxuan Cao, Jiangjiang Qin, Xiangdong Cheng, Zhiyuan Xu

**Affiliations:** ^1^ Second Clinical Medical College, Zhejiang Chinese Medical University, Hangzhou, China; ^2^ Department of Gastric Surgery, The Cancer Hospital of the University of Chinese Academy of Sciences (Zhejiang Cancer Hospital), Institutes of Basic Medicine and Cancer (IBMC), Chinese Academy of Sciences, Hangzhou, China; ^3^ Key Laboratory of Prevention, Diagnosis and Therapy of Upper Gastrointestinal Cancer of Zhejiang Province, Hangzhou, China; ^4^ Zhejiang Provincial Research Center for Upper Gastrointestinal Tract Cancer, Zhejiang Cancer Hospital, Hangzhou, China

**Keywords:** tumour-associated macrophages, gastric cancer, immunotherapy, tumour immune microenvironment, bacteriophage

## Abstract

As one of the deadliest cancers of the gastrointestinal tract, there has been limited improvement in long-term survival rates for gastric cancer (GC) in recent decades. The poor prognosis is attributed to difficulties in early detection, minimal opportunity for radical resection and resistance to chemotherapy and radiation. Macrophages are among the most abundant infiltrating immune cells in the GC stroma. These cells engage in crosstalk with cancer cells, adipocytes and other stromal cells to regulate metabolic, inflammatory and immune status, generating an immunosuppressive tumour microenvironment (TME) and ultimately promoting tumour initiation and progression. In this review, we summarise recent advances in our understanding of the origin of macrophages and their types and polarisation in cancer and provide an overview of the role of macrophages in GC carcinogenesis and development and their interaction with the GC immune microenvironment and flora. In addition, we explore the role of macrophages in preclinical and clinical trials on drug resistance and in treatment of GC to assess their potential therapeutic value in this disease.

## 1 Background

Gastric cancer (GC) is the fifth most common cancer worldwide and the third leading cause of cancer death (1). At present, the main treatment methods for GC are comprehensive therapy mainly based on surgical treatment, including radiotherapy and chemotherapy, targeted therapy and immunotherapy. However, the prognosis of GC remains poor, with a 5-year survival rate of no more than 30% (2). With the development of molecular targeted therapy and immunotherapy, the prognosis of non-small cell lung cancer, breast cancer and other malignant tumours has significantly improved in recent years. Meanwhile, these new treatments have also provided obvious survival benefits for patients with GC.

The tumour microenvironment (TME) consists of different types of cells, including immune cells, stromal cells, small cell organelles, RNA, blood vessels, lymphatic vessels, the extracellular matrix (ECM) and secreted proteins (3). The TME impedes drug access to malignant lesions by restricting blood flow, leading to chemoresistance and suppression of antitumour immune responses (4). As important immune cells in the TME, tumour-associated macrophages (TAMs) are characterized by M2 polarization and support cancer in a variety of ways, such as promoting inflammation and angiogenesis and inducing the epithelial-mesenchymal transition (EMT).

Macrophages are the most abundant inflammatory cells in the TME, with significant heterogeneity and plasticity, and they play a central role in maintaining tissue homeostasis and regulating immune metabolism (5). Crosstalk between macrophages and the TME also plays an important role in promoting tumour progression (6). Recently, there has been growing evidence that immunotherapy targeting macrophages can improve the prognosis of GC patients. Therefore, targeting macrophages is considered to be one of the most promising and effective approaches for the future treatment of GC. In this paper, we review the progress of research on the distribution and polarisation of macrophages and their role in the development of GC, including crosstalk between these cells and the TME. In addition, macrophage-targeted therapies are explored and summarised.

## 2 Origin, classification, activation and polarisation of macrophages in cancer

Macrophages are one of the most important immune cells that maintain the dynamic balance of tissues and maintain homeostasis of the immune system. At the end of the 19th century, Elie Metchnikoff introduced the term “macrophage”, and recently, there have been significant advances in understanding the origin of macrophages. For many years, it was thought that macrophages originated exclusively from monocytes in the bone marrow. However, some of the latest evidence has shown that many macrophages colonise tissues originating from the yolk sac during embryonic development and that these cells are self-renewing into adulthood independently of steady-state monocyte inputs, such as embryonic-derived microglia, adult monocytes and embryonic dual-derived cardiac macrophages (7). Although the origin of macrophages remains controversial, it has been shown that haematopoietic stem cells are not the only source; indeed, other sources, such as yolk sacs, adult monocytes and foetal monocytes, exist.

### 2.1 Classification of tumour-associated macrophages

**Figure 1:**
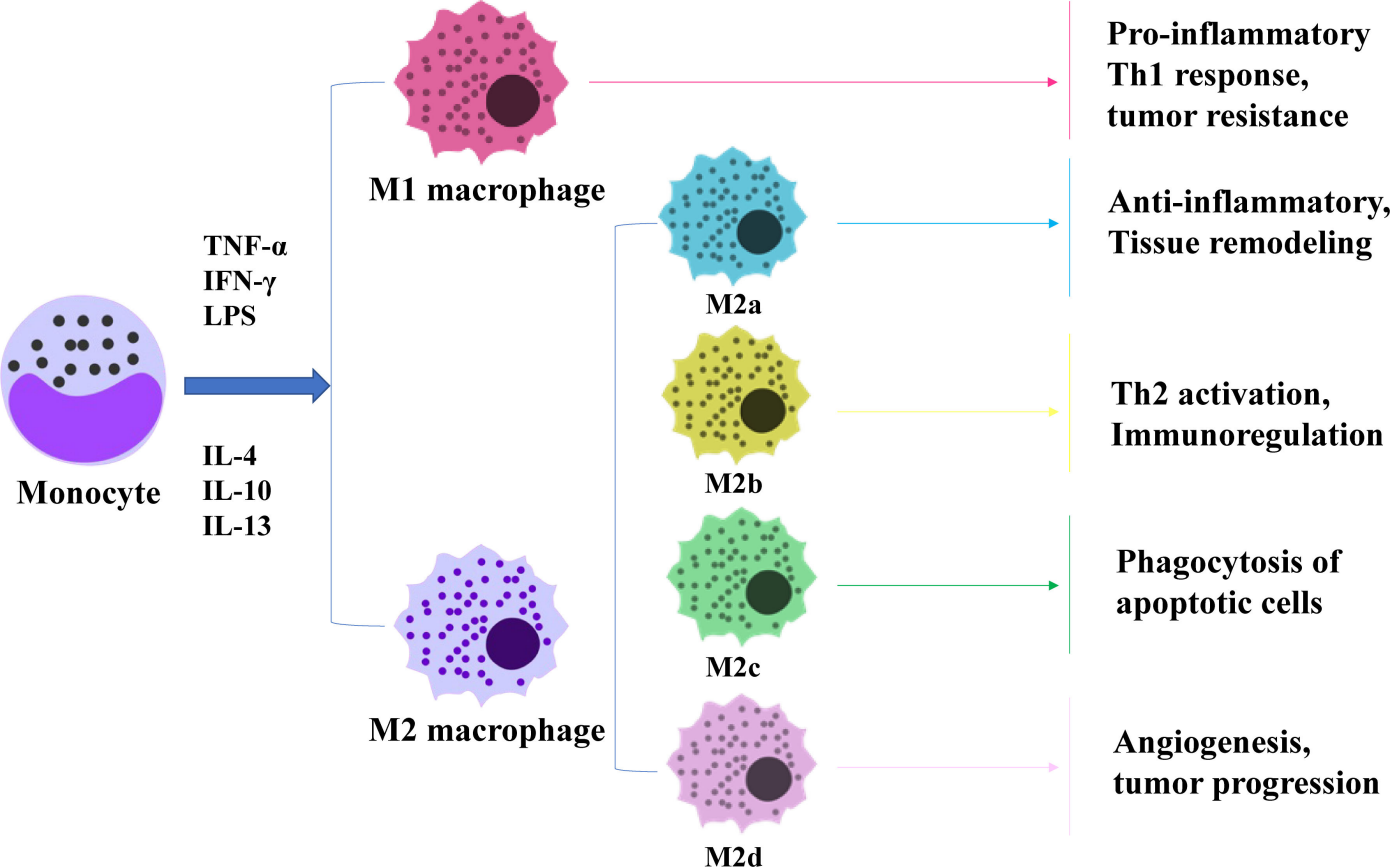
Diagram of macrophage polarization. Macrophages are polarised by TNF-α, IFN-γ and LPS as M1 subtypes and by IL-4, IL-10 and IL-13 as M2 subtypes. M2 macrophages can be subdivided into four subtypes, each performing a different function.

Macrophages are remarkably plastic and heterogeneous, and their phenotype and function are regulated by the surrounding microenvironment. In tissues, macrophages respond to environmental changes by acquiring different functional phenotypes. Tumour-associated macrophages (TAMs) can be divided into two main phenotypes: classically activated (M1) macrophages and alternatively activated (M2) macrophages (8). M1 macrophages normally exhibit a proinflammatory phenotype through secretion of interleukin-1β (IL-1β), interleukin-6(IL-6) and tumour necrosis factor-α (TNF-α) and play a key role in antitumour activity (9). In contrast, M2 macrophages have anti-inflammatory and pro-angiogenic effects, and they can maintain tissue dynamic homeostasis and fibrin production and inhibit antitumour responses similar to those of M1 macrophages (9). Macrophages undergo classical M1 activation (stimulated by Toll-like receptors(TLRs) and interferon-γ (IFN-γ)) or alternate M2 activation (stimulated by IL-4/IL-13) in response to various signals, which reflects the Th1-Th2 polarisation of T cells. However, the “M1/M2” designation does not cover the full range of macrophage phenotypes, and M2 macrophages can be further divided into four distinct subsets: M2a, M2b, M2c and M2d (10) (**Figure 1**). M1 and M2 macrophages have different chemotactic signatures, with M1 macrophages expressing helper T1(Th1) cell chemokines such as chemokine (C-X-C) motif ligand (CXCL)9 and CXCL10 and M2 macrophages expressing chemokines chemokine ligand (CCL)17, CCL22 and CCL24. Chemokines also influence macrophage polarisation, with CCL2 and CXCL4 driving the cells towards an M2-like phenotype (8). In the TME, macrophages can switch from one phenotype to another, and transformation of the two phenotypes provides new ideas for tumour therapy.

TAMs usually exhibit an M2-like phenotype and may have a strong immunoreactive function in the initial stages of cancer; in later stages, the microenvironment is enriched with growth factors and anti-inflammatory mediators such as IL-4, IL-10 and transforming growth factor-β(TGF-β), which induce macrophage polarisation, and the cells thus acquire an M2 phenotype with tumour-promoting functions (9). The M2 phenotype of TAMs not only promotes tumour proliferation but is also associated with poor prognosis in many cancers. For example, it has been reported that the density of M2 macrophages is associated with poor prognosis in patients with renal cell carcinoma and intrahepatic cholangiocarcinoma. Moreover, intraperitoneal TAMs in GC patients with peritoneal metastasis differentiate towards the M2 phenotype and may be involved in tumour proliferation and progression (11).

### 2.2 Regulation of macrophage polarisation and activation in cancer

Inflammation is one of the hallmarks of cancer. One of the features of the TME in GC is chronic inflammation originating from infections, such as *Heliobacter pylori*, which is the strongest single risk factor for GC (12). A large number of connective tissues and abundant immune cell infiltration, including macrophages, myeloid suppressor cells (MDSCs), and regulatory T cells (Tregs), form a highly immunosuppressive TME (4). TAMs are the major infiltrating leukocytes in the TME (13), and TAM infiltration in tumour tissues correlates positively with poor prognosis in GC. TAMs are thought to promote cancer progression by secreting a variety of factors, including inflammatory cytokines, growth factors and protein hydrolases. Mature macrophages can also polarise in response to environmental signals. Macrophage polarisation is defined as an estimate of macrophage activation at a given point in time and space (14). Nevertheless, the distinction between the terms “polarisation” and “activation” is still rather vague, with “polarisation” being used more often in the exclusive field. The two widely known macrophage polarisation programmes are classically activated (M1) and alternatively activated (M2) macrophages, which are induced by different stimuli. Macrophages polarise towards the M1 phenotype in response to factors such as lipopolysaccharide (LPS), IFN-γ, and TNF-α, which play a proinflammatory role and immune function. In contrast, genetic evidence suggests that TH2 cell-derived IL-4 and IL-13 may play a key role in the M2 polarisation of macrophages and their procancer function. In pancreatic cancer models, IL-4 induces substantial histoproteinase activity in TAMs and mediates tumour growth, angiogenesis and invasion *in vivo* (15). It was reported for the first time that GC-derived mesenchymal stem cells (GC−MSCs) significantly induce polarisation and generation of pro-tumour M2-like macrophages by activating the JAK2/STAT3 signalling pathway through secretion of high levels of IL-6/IL-8 (16). Furthermore, various other stimuli, such as antibody immune complexes with endotoxin or IL-1, glucocorticoids, transforming growth factor-β (TGF-β) and IL-10, can produce an M2-like functional phenotype with the same characteristics as IL-4- or IL-13-activated macrophages (17).

In addition, genetics provides a number of transcription factors associated with macrophage polarisation, including signal transducer and activator of transcription 1 (STAT1), signal transducer and activator of transcription 6 (STAT6), interferon regulatory factor 4 (IRF4), interferon regulatory factor 5 (IRF5), and peroxisome proliferator-activated receptor γ (PPARγ). As shown in **Table 1**, there are many regulators of macrophage polarisation. Complete phenotypic mutational loss of M2 polarisation may involve IL-4, IL-13, STAT6, and the key downstream transcription factors that control M2 gene expression, such as IRF4, histone demethylase Jumonji D3 (JMJD3), peroxisome proliferator-activated receptor δ (PPARδ), and PPARγ. Allelic loss-of-function mutations in any of the genes encoding these factors results in complete or substantial loss of M2 polarisation gene expression or marked reduction in M2 macrophages (11). Some factors only regulate M1 or M2 polarisation. The transcription factor Krüppel-like Factor 4(KLF4)induces M2 polarisation by cooperating with STAT6 to induce the M2 genetic programme while inhibiting the M1 phenotype through suppression of M1 targets via isolation of the coactivators required for nuclear factor-k-gene binding (NF-κB) activation (27). In contrast, Krüppel-like Factor 6(KLF6) promotes the M1 phenotype through collaboration with NF-κB and inhibits the M2 phenotype by suppressing PPAR expression, thereby inducing M1 polarisation while inhibiting conversion of macrophages towards the M2 phenotype (31).

**Table 1:** Macrophage activation and polarization regulators.

Protein/ *Gene*	Function	Related factors	Polarisation / activation	Dependency pathways	Note	Ref.
**IL-4**	Inflammatory factor		Induced M2 polarization			(18)
**IL-13**	Inflammatory factor		Induced M2 polarization			/
**IL-6**	Inflammatory factor		Induced M2 polarization	Activation of the JAK2/STAT3 pathway		(19)
**IL-8**	Inflammatory factor		Induced M2 polarization	STAT3 pathway		(16)
***mIR-217***	mIR	IL-6	Inhibiting M2 polarisation	JAK3/STAT2	Inhibition of M2; polarization through IL-6 inhibition.	(20)
**IL-10**	Inflammatory factor		Induced M2 polarization	NF-κB pathway		(21)
**BMP-6**	Growth differentiation factor	IL-10	Defect will induce M2 polarisation	Smad5/STAT3	Belongs to the TGF-β superfactor family; promotes M2 polarization via IL-10.	(22)
**ELK4**	Transcription factor		Induced M2 polarization	KDM5A-PJA2-KSR1	Promoting macrophage M2 polarization through transcriptional activation of KDM5A and regulation of KSR1.	(23)
**STAT3**	Transcription factor		Induced M2 polarization			(22)
**STAT6**	Transcription factor		Induced M2 polarization			(22)
**Fra-1**	Transcription factor	IL-6	Induced M2 polarization		Binding of Fra-1 to the IL-6 promoter leads to increased IL-6 expression and promotes polarization of M2d macrophages.	(19)
**PINK1**	Signal		Induced M2 polarization			(20)
**PTEN**	Phosphatase		Induced M2 polarization			(24)
***mIR-301a-3p***	mIR		Induced M2 polarization	PTEN/PI3Kγ pathway	Induction of macrophage M2 polarization through activation of the PTEN/PI3Kγ signaling pathway.	(24)
**PJA2**	Ubiquitin ligase		Induces M1 polarization Inhibition of M2 polarization	Activation of the JNK/p38 pathway	Ubiquitination reduces KSR1 inhibition of macrophage M2 polarization; induction of MFHAS1 ubiquitination and activation of JNK and p38 pathways; promotes M1 polarization.	(25)
**KLF4**	Transcription factor		Inducing M2 polarisation Inhibition of M1 polarization		The mechanism may be that KLF4 cooperates with STAT6 to induce the M2 genetic program and inhibits M1 targets by isolating co-activators required for NF-κB activation.	(26)
**KLF6**	Transcription factor		Inhibition of M2 polarization Induces M1 polarization		The mechanism may be that KLF6 promotes the M1 phenotype through collaboration with NF-B and suppresses the M2 target by inhibiting PPAR expression.	(27)
**AKT1**	Signal		Induced M1 polarization			(28)
**AKT2**	Signal		Induced M2 polarization			(28)
**INF-γ**	Inflammatory factor		Induced M1 polarization			/
**NF-κB**	Transcription factor		Induced M1 polarization			(29)
**STAT1**	Transcription factor		Induced M1 polarization			(22)
**GM-CSF**	Inflammatory factor		Induced M1 polarization			(30)
***mIR-223***	mIR		Induced M2 polarization	PPARγ/microRNA-223 adjustment shaft		(31)
**TNF-α**	Inflammatory factor		Induced M1 polarization			(32)
**c-Jun**	Transcription factor		Induced M1 polarization		A member of the AP-1 transcription factor, which directly activates cox-1 and indirectly inhibits Arg-1 to regulate macrophage activation.	(33)
**IRF-4**	Transcription factor		Induced M2 polarization		Regulation of M2 gene expression with JMJD3.	(21)
**IRF-5**	Transcription factor		Induced M1 polarization		Regulation of M1 polarization by activation of Akt2.	(34)
**PPARγ**	Transcription factor		Induced M2 polarization			(35)

Moreover, specific factors in the STAT family play an important role in the polarisation of myeloid cell function. In particular, STAT1, STAT3 and STAT6 have been shown to play a major role in transmitting polarisation signals to the nucleus and have distinct functions in macrophage polarisation. STAT1 is activated by M1 macrophage polarisation signals (e.g., INF-γ and LPS), whereas STAT3 and STAT6 are selectively activated by M2 macrophage polarisation cytokines (e.g., IL-10, IL-4 and IL -13) (36). STAT2 and STAT3 induce M2 polarisation; STAT1 is confirmed to induce M1 polarisation. It was shown that in ovarian cancer, miR-217 inhibits tumour-induced M2 macrophage polarisation by targeting IL-6 and regulating the JAK3/STAT3 signalling pathway and that in renal cell carcinoma, BMP-6 induces M2 polarisation through activation of the Smad5/STAT3 pathway by IL-10. Accordingly, the STAT3 pathway may be the major pathway regulating M2 macrophage polarisation (29), and blocking the STAT3 signalling pathway is expected to inhibit M2 macrophage polarisation. In addition, Fra-1, ELK4 and PJA2 have been found to exert regulatory effects on macrophage polarisation and activation. An additional movie file shows this in more detail (see in Tables file. **Table 1**.).

## 3 Effect of macrophages on the proliferation, invasion and metastasis of gastric cancer cells

M2 TAMs promote tumour growth, invasion and metastasis, whereas M1 macrophages inhibit tumour progression by releasing tumour-killing molecules, including reactive oxygen species (ROS) and nitric oxide synthase (iNOS). M2-type macrophages are the dominant macrophages in the TME (37). TAM infiltration correlates positively with tumour cell progression. Many studies have shown that TAMs secrete a variety of cytokines that can promote tumour cell proliferation, including epithelial growth factor (EGF), platelet-derived growth factor (PDGF), transforming growth factor-β1 (TGF-β1), hepatocyte growth factor (HGF) and basic fibroblast growth factor (bFGF) (38). TAMs are an important cellular source of EGF secretion in tumour tissue and have been found to be significantly associated with epidermal growth factor receptor (EGFR) expression in tumour cells and poor prognosis (39). There is an important EGFR/CSF-1R paracrine loop between macrophages and tumour cells. Cancer cells secrete macrophage-colony stimulating factor 1 (CSF-1), which binds to macrophages and promotes polarisation of macrophages towards an M2-like phenotype. CSF-1 also stimulates macrophages to release EGF, thereby promoting proliferation and migration of tumour cells, whereas EGF stimulates secretion of CSF-1 in tumour cells, thus forming a positive feedback loop between tumour cells and macrophages (40). Matrix metalloproteinases (MMPs) secreted by TAMs, which may be indirectly generated through stimulation of tumour vasculature, are also associated with tumour growth. TME metabolic conditions (e.g., hypoxia) induce TAMs to express MMPs, which in the TME can promote EMT, angiogenesis and lymphangiogenesis (41). MMP can also promote invasion and metastasis of tumour cells through the ECM by degrading the basement membrane (BM). In addition, macrophage-derived TGF-β1 enhances the aggressiveness of tumour cells in a mouse model by promoting expression of MMP-9 and increasing the aggressiveness of tumour cells (42). Macrophages activate the JAK/STAT1 signalling pathway by secreting CXCL8 and binding to CXCR1/2 on the GC cell membrane. TAMs promote invasion and migration of GC cells by secreting CCL18, activating the ERK1/2/NF-κB signalling pathway, and increasing expression of Slug and MMP-3. By secreting exosomes, macrophages also regulate malignant transformation and distant metastasis of GC. M2-derived exosomes promote GC migration by activating the PI3K-AKT signalling pathway (43).

## 4 Effect of macrophages on tumour angiogenesis in gastric cancer

Tumour angiogenesis is essential for tumour growth and metastasis. The relationship between macrophages and angiogenesis was first reported by Polverini et al. (44), and many studies have shown that TAMs play an important role in regulating angiogenesis (45). SunderKotter et al. were the first to suggest the possibility that macrophages regulate angiogenesis (46). A growing number of studies have shown that macrophages are involved in the entire process of tumour angiogenesis, including initiation and anastomosis of vascular sprouts, vascular remodelling, vascular maturation and formation of the vascular plexus (47). TAMs secrete a variety of proangiogenic factors, such as vascular endothelial growth factor-A (VEGF-A), TGF-β1, TNF-α, IL-1β, IL-8, IL-10, IL-35, PDGF and FGF-2. Macrophages are the main source of vascular endothelial growth factor (VEGF). The VEGF family includes VEGF-A, VEGF-B, and placental growth factor (PlGF), which are primarily involved in angiogenesis, and VEGF-C and VEGF-D, which regulate lymphangiogenesis (48). The VEGF family has been shown to regulate angiogenesis in a variety of tumours, including GC. It has recently been reported that macrophages play a role in tumour angiogenesis by stimulating microvascular density (MVD), which is involved in formation of human GC vessels (49). Macrophage-derived VEGF-A is one of the most important VEGFs that promotes tumour-associated angiogenesis in GC. Therefore, inhibition of VEGF-A has become a therapeutic strategy for many cancers, including ramucirumab, a monoclonal antibody targeting the major receptor for VEGF-A and has been shown to improve prognosis in second-line treatment of GC (50). Cyclo-oxygen-ase-2 (COX-2) expression in macrophages also regulates angiogenesis. Recent studies have shown that when cocultured with GC cells, M2-polarised macrophages secrete MMP9 via upregulated COX-2 expression, promoting tumour angiogenesis and invasion of GC. Combined treatment with EGFR and COX-2 inhibitors significantly inhibit gastric tumorigenesis in transgenic mice (51). Moreover, hypoxia is considered to be an important stimulus that induces tumour-associated angiogenesis in GC. Newly formed blood vessels in tumours are often disorganised, causing poor regional blood flow and hypoxia. Hypoxia promotes recruitment of proinflammatory macrophages through chemokine secretion. Macrophages induce expression of HIF-1α, which is involved in production of VEGF-A (52). Overall, HIF-1α is considered a useful independent prognostic factor in GC (53), and gastric tumour growth, angiogenesis and angiogenesis can be inhibited by suppressing HIF-1α activity.

**Figure 2:**
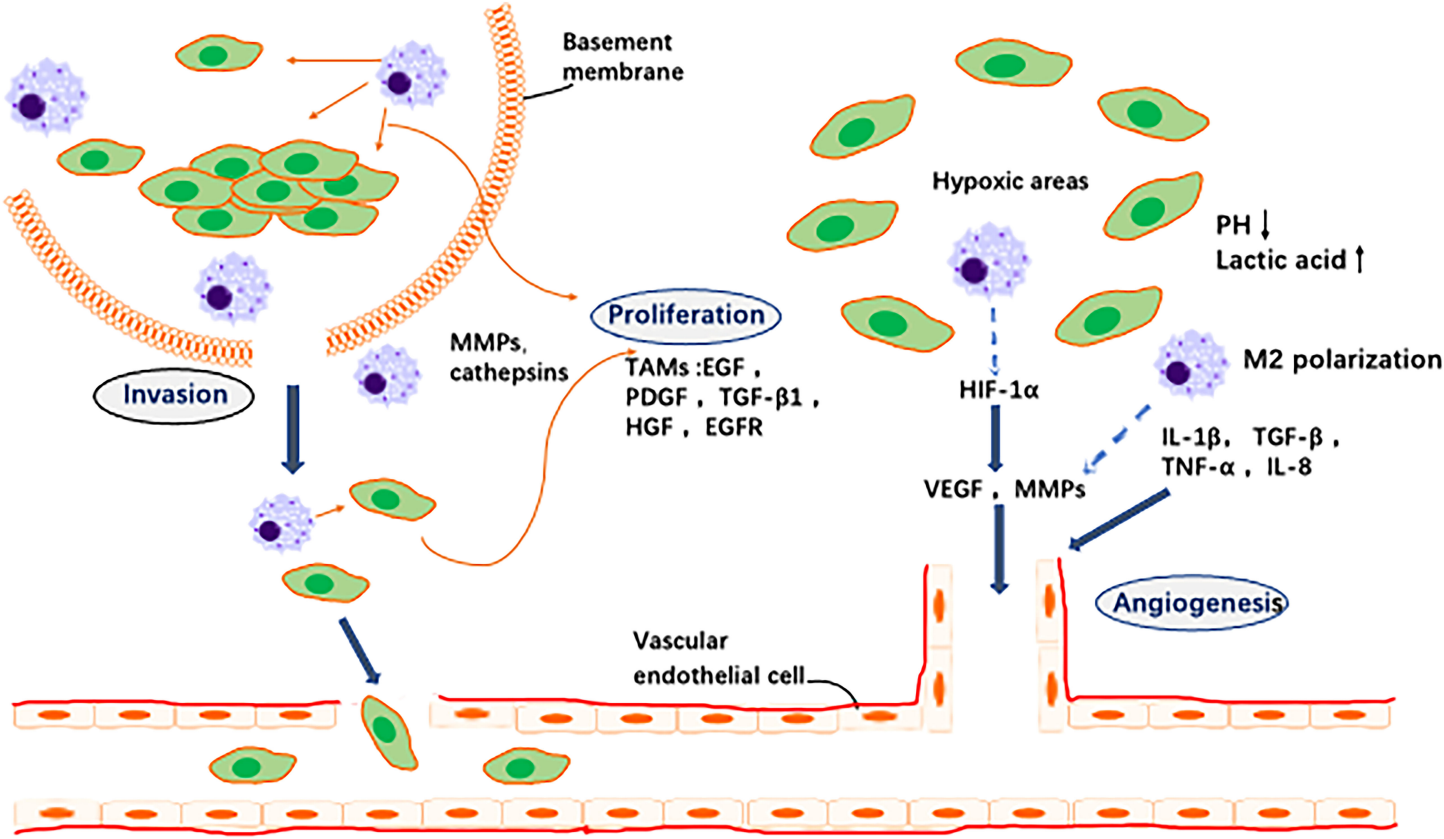
Effects of TAMs on tumor cells and angiogenesis. 1. Invasion: TAMs secrete a variety of proteases that break down the basement membrane surrounding proliferating tumor cells (e.g., ductal carcinoma *in situ* of the breast), thereby contributing to their escape into the surrounding stroma where they exhibit unregulated growth. 2. Angiogenesis: In areas of transient (avascular) and chronic (paroxysmal) tumor hypoxia, macrophages cooperate with tumor cells to induce vascular supply in this region by upregulating a number of angiogenic growth factors and enzymes. These diffuse away from the hypoxic region and, together with other pro-angiogenic stimuli in the tumor microenvironment, stimulate the migration, proliferation and differentiation of endothelial cells in the adjacent vascularised region into new blood vessels.3. Proliferation: a variety of macrophage factors promote the proliferation of tumor cells.

Angiogenesis is a key link in tumour growth and metastasis. TAMs and their secreted cytokines increase vascular permeability, leading to distant metastasis of GC cells. Thus, inhibition of TAMs is an effective measure to inhibit GC angiogenesis and reduce tumour cell migration. In conclusion, TAMs are important regulators of angiogenesis and involved in formation of new blood vessels and remodelling into a coherent functional network. They migrate to hypoxic/necrotic areas of the tumour where vascularisation is necessary for tumour cell survival and are then activated by local signals, such as hypoxia, to synthesise angiogenic regulators. This contributes to formation of new blood vessels and promotes local tumour growth and survival (**Figure 2**).

## 5 Effect of macrophages on the tumour immune microenvironment in gastric cancer

**Figure 3:**
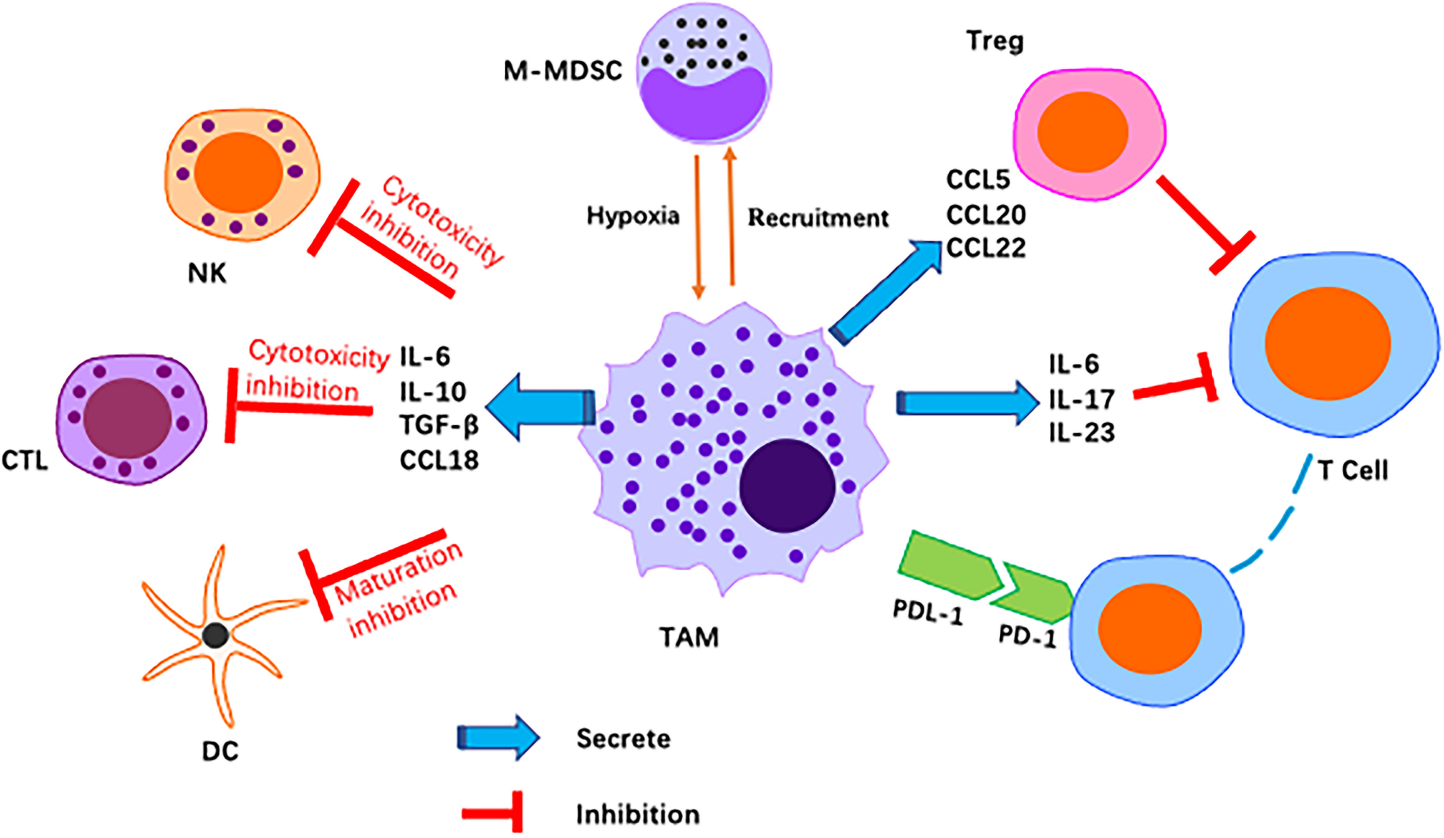
Schematic representation of M2-like macrophages regulation of various immune cell pairs in the tumor microenvironment. Macrophages act as inhibitors/promoters of immune cells by secreting various factors.

The tumour immune microenvironment (TIME) is a complex ecosystem of adaptive and natural immune cells with pro- and antitumour effects (54). The tumour microenvironment contains numerous immune-related cells, mainly macrophages, dendritic cells (DCs), MDSCs, T cells, mast cells and natural killer (NK) cells. All of these factors play a key role in resistance to infection and other diseases (55). M2-like TAMs constitute the main immune cell population present in the TIME of GC. M2-like TAMs suppress immune responses, leading to immunosuppression (56) (**Figure 3**).

T-cell-mediated adaptive immunity is considered to play a major role in antitumour immunity. M2-like TAMs directly and indirectly inhibit cytotoxic T-cell activity. As reported, M2-derived CCL22 regulate T-cell recruitment and may be an important mediator of this recruitment. M2-like TAMs secrete anti-inflammatory cytokines and chemokines, including CCL5, CCL20 and CCL22, that indirectly inhibit T cells and NK cells, causing immunosuppression (57). Programmed death ligand 1 (PD-L1) is a negative costimulatory molecule with a primary function that is thought to involve regulation of T cells. In fact, many studies have demonstrated the critical role of the programmed death 1/programmed death ligand 1 (PD-1/PD-L1) pathway in immune evasion by tumours. The PD-1/PD-L1 axis, as an immune checkpoint, has been shown to block T-cell activation and promote differentiation of CD4+ T cells into Foxp3+ regulatory T cells (Tregs) (58). M2-like macrophage infiltration correlates positively with PD-L1 expression by GC cells (59). Other studies have reported that TMAs induce PD-L1 expression by secreting TNF-α and IL-6 (60). PD-L1+ macrophages form an immunosuppressive microenvironment in GC (61). Metabolic regulation between the TIME and TAMs also has an impact on the immune microenvironment. It has been reported that macrophages inhibit T-cell toxicity by competitive uptake of arginine by T cells (62), and a later study found that arginine is essential for T-cell survival and antitumour immunity (63). This is one of the important reasons for increased TAM accumulation in tumour tissues, namely, inhibition of the antitumour immune response of T cells. Moreover, neuro-oncological ventral antigen 1(NOVA1) inhibition is frequently found in the GC microenvironment, which may be related to immune dysfunction caused by changes in the composition of T cells and macrophages (64).

M2-like TAMs in GC appear to also downregulate expression of IFNγ, TNFα and Ki-67 in NK cells *in vivo* (65). NK cell-derived IFN-γ promotes M1 polarisation of macrophages. Additionally, TNF-α and granulocyte-macrophage colony stimulating factor (GM-CSF) support an inflammatory phenotype in macrophages. Hence, NK cells play an important role in maintaining the proinflammatory phenotype of TAMs, and TAMs have the capacity to suppress NK cells (66). Some studies have suggested that TAMs suppress the activity of NK cells by secreting PGE2 and TGF‐β in the TME (67).

## 6 Interaction between macrophages and the bacterial flora in gastric cancer

The human gastrointestinal tract contains approximately 10-100 trillion bacteria, comprising approximately 500-1500 different species (68). As the largest and most complex ecosystem in the human body, the intestinal flora and its thousands of metabolites influence almost every aspect of the host’s physiological activity (69). Although most microbes are engulfed and killed by macrophages, some bacteria live inside macrophages as opportunistic residents and use them to replicate (70). M1 macrophages can be controlled by microbial stimuli, including intracellular bacteria, to support cytotoxic activity and infection resistance. Some bacteria promote M2 polarisation or interfere with M1 polarisation to thrive in the microenvironment. To avoid cytotoxic effects and evade the cellular immune response, microbes such as *Fusobacterium nucleatum* possibly promote M2-polarised macrophages (71). When host pathogens interact, live bacteria or their components often trigger innate immune cell reactions and cause immune cells, such as macrophages, to migrate towards tumours (70). Data show that microbial pathogens play a carcinogenic role in gastrointestinal tumorigenesis (72). Wan et al. demonstrated that these bacteria increase secretion of IL-6 and TNF-α by activating TAMs, promoting EMT in colorectal cancer (73). Several bacteria are known to interact with macrophages in tumours, such as *H. pylori*, which has been shown to affect gastric tumour growth.

### 6.1 Interaction between the bacterial flora and macrophages in gastric cancer

#### 6.1.1 Helicobacter pylori

*H. pylori* was first discovered in 1982 in the stomach of patients with peptic ulcers (74). *H. pylori* infection is an important risk factor for GC, causing inflammation and damage (75–78). Macrophages detect the presence of pathogen-associated molecular patterns (PAMPs) in *H. pylori* through pattern recognition receptors (PRRs) such as Toll-like receptors (TLRs) and NOD-like receptors (NLRs). Studies have shown that inflammation caused by *H. pylori* infection is associated with expression of TLR4 and TLR9. Furthermore, TLR9 plays a major role in the inflammation occurring in GC (70). Once PAMPs are recognised, TLRs induce polarisation of TMAs by activating activator protein (AP)-1, interferon regulatory factor (IRF) and NF-kB, which promote expression of inflammatory mediators such as TNF-α, IL-1, IL-2, IL-6, IL-8, IL-12 and INF-γ (79). NOD1 detects bacteria (bacterial peptidoglycan particles) and mediates production of inflammatory factors, and loss of NOD1 can accelerate stomach carcinogenesis in a mouse model. The wild-type phenotype of macrophages rapidly changes from M2 to M1 after *H. pylori* infection. NOD1-deficient macrophages also exhibit a more pronounced M2 phenotype (80). Macrophages kill phagocytic pathogens through oxygen-dependent and nondependent mechanisms, and specific recognition of human leukocyte antigen (HLA)-II-peptide complexes on the surface of phagocytes by Th cells can enhance their killing potential. Recent studies have found that macrophages infected with *H. pylori* exhibit strongly reduced expression of HLA-II molecules on the plasma membrane, which compromises bacterial antigen presentation to Th lymphocytes (81). Codolo et al. demonstrated that *H. pylori* hampers HLA-II expression in macrophages, activated or nonactivated by IFN-γ, by downregulating expression of the class II major histocompatibility complex transactivator (CIITA) (81). Another study found that loss of MMP7 boosts M1 macrophage polarisation and exerts a restrictive role on *H. pylori*-induced gastric injury as well as development of premalignant lesions by suppressing M1 macrophage polarisation (82).

Stomach cancer caused by *H. pylori* infection is associated with its virulence (83). Cytotoxin-associated gene A (CagA) and vacuolar toxin A (VacA) are the most intensively studied virulence factors in *H. pylori* infection. Injection of CagA into host gastric epithelial cells is involved in dysregulation of cell proliferation and apoptosis by interfering with the PI3K/Akt, MEK/ERK and Wnt/β-catenin signalling pathways (84). In addition, it has been shown that CagA induces an inflammatory response through activation of the NF-κB pathway (85). *H. pylori* virulence factors are involved in the host immune response. For instance, release of inflammatory mediators activates the Th1/Th17 cell response and stimulates production of TNF-α, IL-17 and INF-γ (86), inducing macrophage polarisation towards the M1 phenotype.

**Figure 4:**
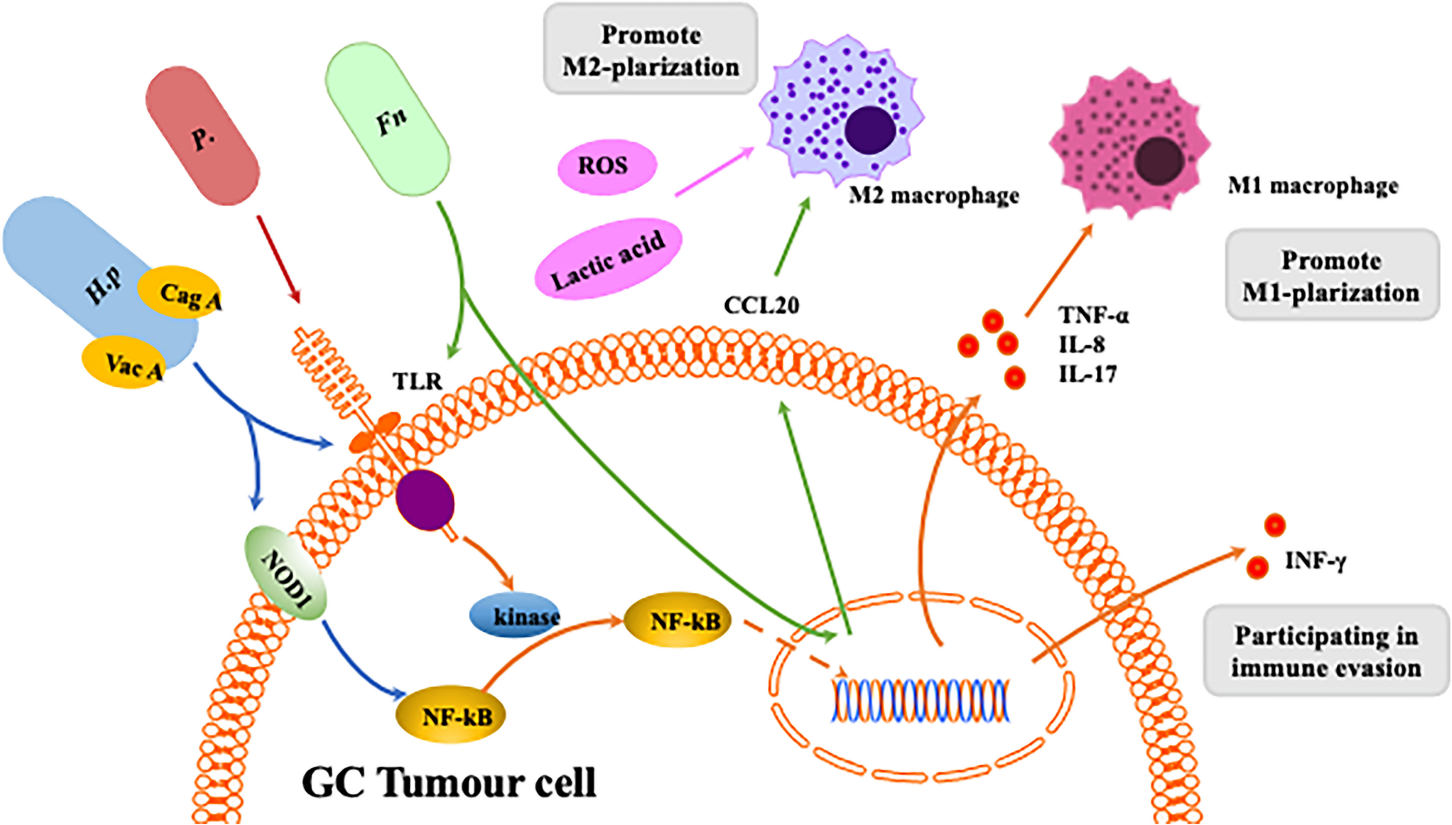
Interaction between homogeneous flora and macrophages in gastrointestinal tumors. The Helicobacter pylori virulence factors CagA and VacA are involved in the host immune response.CagA induces an inflammatory response through activation of the NF-κB pathway.CagA is associated with NF-κB-induced responses following NOD1 activation. VacA toxin suppresses host immunity by inhibiting T cell activation and inducing regulatory T cells. Release of inflammatory mediators induces macrophage polarization towards M1. Fn can induce macrophages to differentiate into M1 or M2 phenotypes through different pathways. P. anaerobius and microbial metabolites like ROS and lactic acid can induces macrophage polarization towards M2.

There is evidence that the NF-κB signalling pathway is involved in *H. pylori*-associated gastric tumorigenesis (87) (**Figure 4**). Specifically, *H. pylori* infection increases NF-κB activity and enhances nuclear heterodimer p50/p65 and homodimer p50 translocation in transformed gastric epithelial cells. *H. pylori* also activates the NF-κB pathway and induces production of proinflammatory cytokines such as IL-8 and IL-17 (88). Macrophages detect PAMPs from *H. pylori* through cellular PRRs. TLRs and NOD1 are common PRRs involved in activating the NF-κB signalling pathway (89). The virulence factor CagA has been identified as relevant to NF-κB-induced responses following NOD1 activation (90), and Lu et al. reported that ROS and HIF-1α regulate *H. pylori*-mediated macrophage polarisation via the Akt/mTor pathway (91).

#### 6.1.2 Fusobacterium nucleatum

*Fn*, a gram-negative bacterium, is a common component of the oral microbiota (92) and is considered a potential risk indicator for inflammation-associated colorectal cancer (CRC) (93). *Fn* was also found to be enriched in GC tissue (T-GC) and the paracancerous mucosa (N-GC) (94). Recently, *Fn* infection has been reported to increase TAM infiltration and be involved in mediating M2-MJ polarisation, promoting CRC progression (93). In CRC patients, *Fn* promotes macrophage infiltration by activating tumour-derived CCL20 while inducing M2 macrophage polarisation and enhancing CRC metastasis through the miR-1322/CCL20 axis (95). However, there are few articles to date about the connection between *Fn* and macrophage polarisation in GC.

#### 6.1.3 Propionibacterium acnes

*Propionibacterium acnes* is a gram-positive anaerobic bacterium that extensively colonises human skin, and recent studies have shown that *P. acnes* may be present in the stomach. *P. acnes* is abundant in GC tissues and promotes progression of GC by promoting M2 polarisation of macrophages through TLR4/PI3K/Akt signalling (96). Nevertheless, little is known about *P. acnes* because of the late discovery of the pathogenicity of this bacterium.

### 6.2 Effect of microbial metabolites on macrophages

Intestinal bacteria produce different metabolites that influence progression and development of gastrointestinal tumours (97). The gut microbiota produces butyric acid, which differentiates Tregs and IL10 to generate T cells through activation of histone deacetylase inhibition (HDACi), interaction with G protein-coupled receptor 43 (GPR43) and IL10 upregulation (98). TLRs can promote development of gastrointestinal tumours through activation of the STAT3 and NF-kB signalling pathways (78). Furthermore, activation of the STAT3 signalling pathway upregulates TLR2 expression in gastric epithelial cells and promotes development of gastric tumours in mice. Single-chain fatty acids, such as butyrate, produced by the gut microbiota may inhibit cancer and inflammation by blocking activation of the NF-kB signalling pathway and by inducing differentiation of IL10-producing T cells and regulatory T cells (99). Many bacteria (e.g., Lactobacillus, Neisseria, Staphylococcus, Haemophilus, Clostridium and Willebrandia) promote GC by stimulating production of N-nitroso compounds (NOCs) (99), and high levels of lactic acid bacteria are found in GC patients. The above bacteria may increase risk of GC through several mechanisms, such as increased production of ROS, NOCs and lactate and immune tolerance, in addition to inducing EMT. The lactic acid produced by Lactobacillus is a powerful source of energy for cancer cells, and lactic acid induces M2 phenotype polarisation of macrophages (53). It also has a regulatory role in carcinogenesis, such as tumour angiogenesis and metastasis (100). ROS, mainly derived from superoxide anions (O2-), hydrogen peroxide (H2O2) and hydroxyl radicals (OH-), are synergistic or independent regulators of cellular signalling in response to different environmental stimuli rather than merely harmful byproducts of cellular metabolism (101). In fact. ROS have been reported to be involved in DNA repair, the cell cycle, cell differentiation, chromatin remodelling, self-renewal and other cellular processes (102). ROS also play a critical role in regulation of macrophage polarisation. Decreased levels of ROS inhibit the M1 phenotype and promote a shift in macrophage polarisation towards the M2 phenotype (103).

### 6.3 Lipopolysaccharide

The gut microbiota interacts with the immune response, mainly through the antigenicity of its own components and metabolites produced by the breakdown of nutrients in food. LPS is a component of the cell wall of gram-negative bacteria in the gut. LPS stimulates small intestinal epithelial cells (IECs) through the cell surface TLR pathway, causing phosphorylation of interleukin receptor associated kinase(IRAK)and multiple antigen peptide(MAP), increasing IL-8 expression and eliciting an immune response (104). Macrophages are among the most abundant cells in the microenvironment of colon cancer, and there is a close association between monocytes, macrophages and the intestinal microbiota. In a mouse model of colitis-associated tumorigenesis, monocyte-like macrophages (MLMs) were selectively increased, and inflammatory cytokine secretion was enhanced in the early stages of colitis-associated carcinoma. MLM accumulation is regulated by CCL2 expression in colonic epithelial cells, as influenced by bacterial-derived LPS. Additionally, LPS stimulates IL-1β production by macrophages and induces activation of IL-17-producing helper T cells to promote inflammation. The gut microbiota appears to regulate MLM accumulation in a chemokine-dependent manner using endotoxin as a trigger and generating a precancerous inflammatory environment that promotes tumorigenesis (105). LPS induces COX-2 and IL-1β expression in MLMs, an effect that can be reversed by inhibition of TLR4. The combination of endotoxin, MLMS and inflammatory cytokines may increase intestinal permeability and allow for excessive release of commensal bacterial products, which also promotes differentiation of M2 macrophages (106).

In summary, the GC microflora can modulate macrophages and enhance gastric tumour development by suppressing antitumour immunity, activating oncogenic signalling pathways and producing protumour metabolites.

## 7 Role of macrophages in treatment of gastric cancer

### 7.1 Effect of macrophages on drug resistance in gastric cancer

Currently, drug resistance is one of the most important risks for treatment failure in GC. In general, development of GC therapeutic resistance has been strongly associated with genetic mutations, metabolic reprogramming, GC stem cells, EMT and a hypoxic TME. Recently, there has been growing evidence that alterations in the TME are an important cause of tumour drug resistance (107). Many studies have shown that TAMs induce resistance to tumour therapy by promoting EMT and tumour angiogenesis, suppressing T-cell function, and secreting inflammatory cytokines and chemokines, among others.

#### 7.1.1 Chemotherapy

Cisplatin-based chemotherapy is commonly used for advanced GC treatment, and cisplatin resistance has been shown to be associated with M2 macrophages. Notably, TAM-derived exosomes are involved in this process. Zheng et al. demonstrated that M2-polarised macrophages promote resistance of GC cells to cisplatin (DDP) and that M2 macrophage-derived exosome miR-21 (M2-exo miR-21) reduces chemosensitivity to cisplatin (108). Recent studies have also demonstrated the role of the long-stranded noncoding RNA (lncRNA) CRNDE in the generation of cis-DDP resistance in GC cells via M2-exo derived from M2 macrophages (109). M2-type macrophages, induced by Yes-associated Protein1(YAP1)-overexpressing GC cells, enhance tumour cell resistance to 5-fluorouracil(5-FU) by secreting CCL8 and activating phosphorylation of the JAK1/STAT3 signalling pathway (110). Furthermore, chemokines produced by TAMs influence doxorubicin resistance. M2-like TAMs in the TME secrete CXCL12 and induce drug resistance via the CXCL12/CXCR-4 axis (111, 112). In xenograft mice with prostate cancer, doxorubicin treatment induces upregulation of CXCR4 in cancer cells and artificially stimulates CSF-1, which in turn activates secretion of CXCL12 by TAMs, resulting in drug resistance (113). In addition to the abovementioned effects of resistance to several common chemotherapy drugs, CSF-1 secreted by stomach cancer cells promotes recruitment of TAMs, which is thought to contribute to chemoresistance. Expression of CSF-1 or CSF-1R correlates positively with GC tissue VEGFA or Fms-associated tyrosine kinase 1 (FLT1) expression. Treatment with recombinant human CSF-1 promotes proliferation, migration and loss of apoptosis resistance in GC cell lines (114). Extensive research has revealed a close correlation between TAMS and chemoresistance, and development of GC chemoresistance is associated with polarisation of TAMs, production of cytokines, activation of related signalling pathways and recruitment of TAMs. These studies provide new targets and strategies to address drug resistance in GC clinical treatment.

#### 7.1.2 Targeted therapy

In clinical practice, the current therapeutic targets for GC are mainly HER2 and EGFR and anti-vascular therapy. However, the mechanisms of resistance to such targeted drugs (e.g., trastuzumab, bevacizumab, and ramorumab) are not well understood. As described above, TAMs express VEGF-A, which activates Src signalling in tumours, promotes tumour angiogenesis and tumour growth and is thus involved in resistance to ramucirtumab treatment. Trastuzumab’s mode of action includes inhibition of HER2-mediated cell signalling (115), antibody-dependent cytotoxicity (ADCC) and complement-dependent cytotoxicity (CDC). ADCC is an important aspect of the antitumour efficacy of HER-2-targeted monoclonal antibodies (MABs) (116). TAMs affect normal functioning of ADCC through its surface FCγ receptor (FCγR), leading to drug resistance. Recent studies have shown that bevacizumab treatment induces tumour cells to express CD47 and HIF-1, which can promote macrophage synthesis and polarisation towards the M2 type (117), promoting tumour angiogenesis and causing bevacizumab resistance in GC.

### 7.2 Treating gastric cancer by reversing polarisation and promoting activation

**Figure 5:**
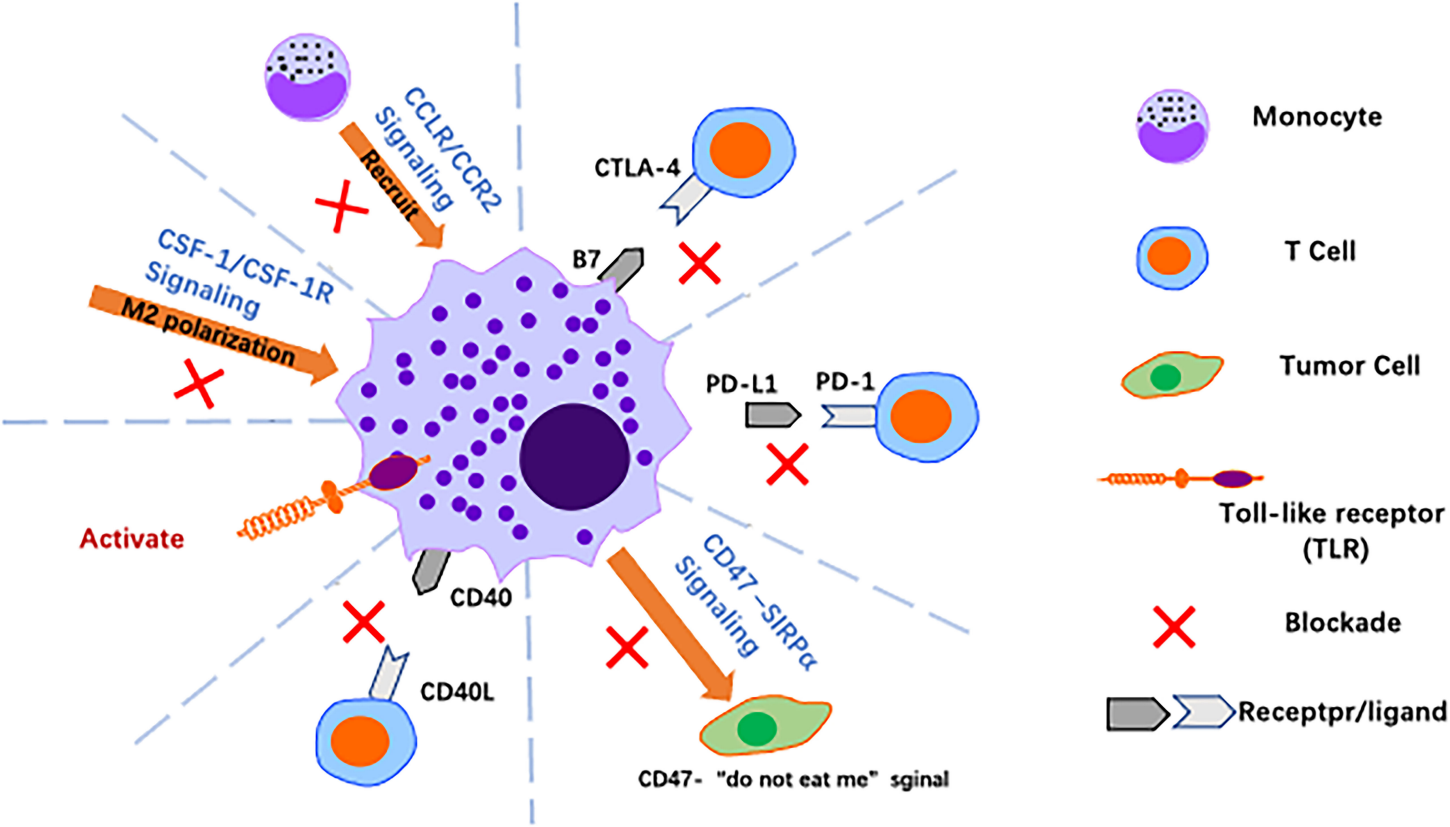
Harnessing macrophages for cancer immunotherapy. Over the past decade, significant advances have been made in cancer immunotherapy using macrophages, with strategies such as reversing polarisation, promoting activation of macrophages and blocking immune checkpoints to alter the tumor immune microenvironment.

In the era of immunotherapy, there is an increasing need to identify new therapeutic strategies to personalise treatment approaches and overcome resistance to checkpoint inhibition. As TAMs are involved in the entire process from tumour initiation to distant metastasis, including the inflammatory response, tumorigenesis, angiogenesis, invasion, immune evasion, metastasis and chemoresistance, targeting TAMs has become a hot research topic in GC immunotherapy. The CCL2/CCR2 axis is critical for macrophage recruitment in a variety of cancers, and the CSF-1/CSF-1R axis is involved in macrophage activation regulation, leading to metastasis of cancer cells. Inhibiting the CSF-1/CSF-1R axis effectively inhibits progression of hepatocellular carcinoma cells (118). CTLA-4, PD-1 and PD-L1 ligands are expressed on both M2-like TAMs and cancer cells, and great success in the treatment of melanoma has recently been achieved through targeting of these molecules (119). Blocking these checkpoints may also inhibit GC. In preclinical models, dual angiopoietin-2/vascular endothelial growth factor (Ang-2/VEGF) bispecific antibodies showed significant antitumour activity and reprogrammed TAMs from an M2 protumour phenotype to an M1 antitumour phenotype (120). Therefore, targeting TAMs may be complementary to current antiangiogenic therapy for GC (**Figure 5**). Some relevant studies on immunotherapy targeting macrophages are detailed below.

The CCL2/CCR2 axis. In GC, the CCL2/CCR2 axis is closely associated with TAM infiltration into tumour tissue and with the clinical prognosis of patients (121). The CCL2/CCR2 axis plays a central role in macrophage-related functions, and it promotes GC progression by regulating tumour-associated inflammation and M1/M2 macrophage polarisation, promoting TAM recruitment and providing antiapoptotic or angiogenic signals (e.g., vascular endothelial growth factor) to tumour cells in the TME. Nywening et al. found that FOLFIRINOX combined with PF-04136309, an inhibitor of CCR2, was able to block the CCL2/CCR2 axis and reduce TAMs without increasing drug toxicity (122). A phase 1b study by NOEL also showed that the combination of NAB-paclitaxel, gemcitabine and PF-04136309 reduced infiltration of TAMs in PC and CD14+CCR2+ inflammatory monocytes in peripheral blood (123). These results suggest that blocking CCL2/CCR2 is a promising approach to reduce TAM infiltration and inhibit tumour progression. Regardless, the anti-CCL2 monoclonal antibody carlumab (CNTO 888) failed to demonstrate a clinical benefit in phase 1 and phase 2 clinical trials in solid tumours (NCT00992186) and metastatic prostate cancer (NCT00537368) due to its inability to reduce serum CCL2 levels.

The CSF-1/CSF-1R axis. The CSF-1/CSF-1R axis is also critical for induction of M2-like polarisation and is involved in production of an immunosuppressive TME. This signalling axis is a potent target for reversing the macrophage phenotype from pro-tumour to antitumour (124). CSF-1R is a tyrosine kinase receptor, and the humanised monoclonal antibody RG7155 (emactuzumab) blocks activation of CSF1R. In mouse tumour models and cancer patients, RG7155 treatment reduced macrophage infiltration into tumours and increased CD8/CD4 T-cell ratios in patient tumour biopsies (40) (NCT01494688). The small molecule PLX3397 is a CSF-1R inhibitor that can be administered orally. PLX3397 penetrates the blood−brain barrier and was tested in a phase II study in patients with recurrent glioblastoma. The drug was well tolerated and enhanced the efficacy of radiation therapy, suggesting that combined inhibition of CSF-1R may improve the efficacy of radiation therapy for glioblastoma (125). In a mouse model of liver cancer, gene expression profiles of TAMs showed that these cells from PLX3397-treated tumours favoured an M1-like phenotype compared to those from vector-treated tumours. Further studies showed that tumour cell-derived CSF-1 protect TAMs from depletion by PLX3397 (118). In conclusion, CSF-1R blockade slows tumour growth by altering polarisation rather than by depleting TAMs, and targeting the CSF1-CSF-1R axis may provide a new strategy for treatment of GC.

Toll-like receptor agonists. TLRs are natural immune pattern recognition receptors that play an important role in activating the natural immune response to their agonists. In a mouse model of mammary tumours, intratumor injection of TLR7 and TLR9 agonists increased monocyte infiltration and repolarisation of macrophages in tumours; similar results were obtained with agonists of TLR7 and TLR8 (3 M-052), which induced repolarisation of macrophages and increased tumour-killing activity against melanoma. With regard to preclinical models, the TLR7 ligand imiquimod is the only TLR agonist approved for clinical use that has shown antitumour activity in basal cell carcinoma, melanoma and cutaneous metastasis of breast cancer (126).

Anti-CD40 antibody. CD40 is a receptor of the TNF receptor superfamily and is expressed by APCs such as monocytes, macrophages, DCs and B cells. The natural ligand for CD40 is CD40L, which is mainly expressed by CD4+ T cells, basophils and mast cells. The CD40-CD40L interaction upregulates expression of MHC molecules and production of proinflammatory cytokines such as IL-12, promoting initial CD4+ and CD8+ T cells to become helper T cells and cytotoxic cells, respectively. Agonistic anti-CD40 antibodies exert tumour-suppressive effects in tumour-bearing mouse models, a result that holds promise for development of clinically relevant anti-CD40 antibodies. In addition, TAM combined with CD40 agonists and anti-CSF1R antibodies results in TAM reprogramming prior to its depletion (127).

Targeting macrophages effectively alleviates malignant disease by reducing TAM infiltration, reverting M2 types to M1 types, and depleting M2-polarized TAMs, among other mechanisms. A growing number of studies have shown it to be a promising avenue for treatment of tumours.

### 7.3 Treatment of gastric cancer through modulation of immune checkpoints

Tumour immunotherapy with immune checkpoint inhibitors (ICIs) has recently shown clinical activity in several types of cancer and can provide survival benefits as monotherapy or in combination with other types of immunotherapy or conventional chemotherapy. Cytotoxic T lymphocyte-associated protein 4 (CTLA-4) and PD-1 are common inhibitory checkpoints expressed on activated T cells, and the CD47-SIRPα axis is involved in phagocytosis of tumour cells by macrophages (128). The State Drug Administration (SDA) has recently approved Sindbio’s PD-1 inhibitor injection Daboxu as first-line treatment for unresectable locally advanced, recurrent or metastatic adenocarcinoma of the stomach and gastroesophageal junction, targeting macrophages and providing a new strategy for cancer immunotherapy through immune checkpoint blockade.

### 7.4 CD47–SIRPα

Blocking the CD47–SIRPα axis induces phagocytosis of tumour cells by macrophages. Signal-regulated protein alpha (SIRPα) on macrophages is linked to CD47, a “do not eat me” signal on cancer cells that prevents macrophages from phagocytosing cancer cells, thus allowing immune evasion (129). The CD47-blocking antibody HU5F9-G4 (hereafter referred to as 5F9) is a macrophage ICI that blocks CD47, induces tumour cell phagocytosis and has therapeutic efficacy. Valerius and colleagues investigated use of 5F9 in combination with rituximab in patients with lymphoma and showed that 5F9 blocked the CD47–SIRPα interaction, enhancing tumour cell phagocytosis by macrophages (130). In another phase 1b study, the antibody 5F9 synergised with rituximab to eliminate B-cell non-Hodgkin’s lymphoma cells by promoting macrophage-mediated antibody-dependent cytophagy, and 5F9 in combination with rituximab showed good activity in patients with aggressive and inert lymphomas (131).

The application of nanotechnology provides new tools for antitumour immunotherapy. Cancer cells induce macrophage differentiation to the M2 phenotype by the colony-stimulating factor, and produce immune evasion through the CD47–SIRPα axis. Genetically engineered cell membrane-encapsulated magnetic nanoparticles (gCM-MNS) can disable both mechanisms. The gCM shell highly expresses the SIRPα variant, which has a remarkable affinity for effectively blocking the CD47-SIRPα pathway, and the MN core promotes M2 TAM repolarisation and synergistically triggers a robust macrophage immune response. In addition, the gCM shell protects MNs from immune clearance; in turn, the MN core magnetically directs gCMs into tumour tissue, effectively promoting their circulation and tumour aggregation (132). Multiple clinical trials have assessed inhibitors of CD47-SIRPα in digestive tract tumours. PT886, a novel bispecific antibody targeting Claudin 18.2 and CD47, is currently undergoing a phase I trial (NCT05482893) in advanced adult patients with gastric, gastro-esophageal junction adenocarcinoma, and pancreatic cancers. This trial aims to evaluate the safety, tolerability, pharmacokinetics (PK), and preliminary efficacy of PT886 in patients with advanced or refractory cancers. Additionally, a prospective study is proposed to utilize patient-derived organoids from colon cancer biopsies to investigate the impact of CD47-SIRPα inhibitors on the tumour immune microenvironment, with an anticipated completion date of 9 January 2027 (NCT05955196).

### 7.5 PD-1/PD-L1

PD-1 is an immune checkpoint receptor that is often upregulated on activated T cells and induces immune tolerance. Tumour cells often overexpress its ligand PD-L1 and thus escape the immune system. Monoclonal antibodies blocking PD-1/PD-L1 have shown significant clinical efficacy in patients with a variety of cancers, including melanoma, colorectal cancer, non-small cell lung cancer and Hodgkin’s lymphoma. Studies have shown that both mouse and human TAMs express PD-1 and that PD-1 expression by TAMs correlates negatively with phagocytosis of tumour cells (133). *In vitro* and *in vivo* models were used to study the effect of anti-PD-L1 antibody treatment of macrophages on the TAM response. Treatment of mice and human macrophages with PD-L1 antibody increased proliferation, survival and activation of spontaneous macrophages (costimulatory molecule expression, cytokine production), and in tumour-bearing RAG mice, TAMs were observed to upregulate costimulatory molecule expression and reduce tumour growth (134). These findings suggest that PD-L1 antibody therapy is significantly effective at treating tumours with PD-1/PD-L1 ICIs.

In a meta-analysis, the level of PD-L1 expression correlated with the overall survival (OS) of GC (HR = 1.46, 95% CI = 1.08~1.98, P = 0.01, random effect) (135). Another Mate analysis reported that ICI therapy provided a modest survival benefit, with an objective efficiency rate of 12.0% and a disease control rate of 34.7%; in particular, anti-PD-1 therapy improved OS at 12 and 18 months and prolonged the duration of response. In advanced GC, anti-PD-1/PD-L1 therapy was more effective in subgroups with PD-L1 positivity, high MSI, EBV positivity or high mutational load (136).

PD-L1 inhibitors such as pembrolizumab, nivolumab and avelumab are currently approved for marketing and have shown promising results in clinical studies of gastrointestinal tumours (GIT). In a multicentre, open-label phase Ib clinical cohort study, pembrolizumab was used to treat 39 patients with PD-L1-positive recurrent, metastatic gastric or gastroesophageal adenocarcinoma. The results showed partial remission in 8 patients and treatment-related grade 3-4 adverse events in 5, with no treatment-related deaths (137). In a phase III study, 493 Asian GC patients with advanced postchemotherapy were randomised in a double-blind clinical trial to nivolumab treatment and placebo groups, with results representing prolonged OS (HR=0.63, 95% CI: 0.51-0.78, P<0.0001), suggesting that nivolumab may be a new treatment option for heavily pretreated patients with advanced GC (138). Avelumab, an anti-PD-L1 human IgG1 monoclonal antibody, blocks the interaction of PD-L1 with PD-1 and CD80 molecules and has been examined in treatment of advanced GC with avelumab versus third-line chemotherapy (139). Overall, combination of PD-1/PD-L1 inhibitors with chemotherapeutic agents is more beneficial than monotherapy for patients with advanced GC.

An open-label, single-arm, phase 2 trial evaluated the combination of lenvatinib plus pembrolizumab in patients with advanced GC. Twenty-nine patients were enrolled for first- or second-line therapy, and 20 (69%, 95% CI 49-85) achieved objective remission. No grade 4 treatment-related adverse events, serious treatment-related adverse events or treatment-related deaths occurred (140). Furthermore, a phase 3 CheckMate 649 study evaluated first-line PD-1 inhibitor therapy for gastric/GEJ/oesophageal adenocarcinoma. There was a significant improvement in OS and a benefit in PFS with nivolumab + chemotherapy compared with chemotherapy in PD L1 CPS ≥ 1 and in all randomised patients. Grade 3 to 4 treatment-related adverse events occurred in 462 (59%, nivolumab plus chemotherapy) and 341 (44%, chemotherapy) of all treated patients (141). Thus, PD-1/PD-L1 monoclonal antibodies in combination with chemotherapy have shown promising antitumour activity and an acceptable safety profile in patients with advanced GC.

The roles of the PD-1/PD-L1 signalling pathway in the polarization of tumour-associated macrophages (TAMs) have received considerable attention in recent years. Several studies have indirectly highlighted the impact of PD-L1 expression in promoting the M2 polarization of TAMs through inhibition tests. For instance, Xiong and colleagues have reported that anti-PD-L1 treatment can remodel the TAM compartment in responsive tumour models towards a more proinflammatory phenotype, primarily by increasing IFN-γ levels (142). In a recent study, Tichet et al. evaluated the effects of the engineered immunocytokine PD1-IL2v in a mouse model of *de novo* pancreatic neuroendocrine cancer, which is resistant to checkpoint and other immunotherapies (143). The study revealed that the treatment targeting anti-PD-L1 had a selective impact on the phenotype and function of tumour-associated macrophages (TAMs). This treatment induced a polarization of TAMs towards a pro-inflammatory and antigen-presenting-cell (APC)-like phenotype. This change in phenotype has the potential to enhance T cell recruitment, as demonstrated by the analysis of myeloid cells using single-cell RNA sequencing (scRNA-seq). Notably, the combination treatment of PD1-IL2v plus anti-PD-L1 showed an enrichment of IL6, TNFα, and inflammatory signalling pathways compared to PD1-IL2v alone. This enrichment was observed in both the macrophage-2 and dendritic cell (DC) clusters (143). However, the mechanism by which PD-L1 overexpression promotes TAM polarization towards the M2 phenotype and the signalling pathways involved in its regulation have not been thoroughly examined. In a study by Pang et al., exosomes from oral squamous cell carcinoma (OSCC) cells were investigated, revealing that PD-L1 induced M2 macrophage polarization through the CMTM6/ERK1/2 signalling pathway. Additionally, the study found that knockdown of CMTM6 in OSCC cells suppressed M2 macrophage polarization while downregulating PD-L1 expression (144). Hartley et al. conducted a study on melanoma and demonstrated that PD-L1 can modify the phenotype of TAMs by delivering a constitutive negative AKT/mTOR signal, resulting in an immune-suppressive cell (M2-like) phenotype of TAMs (134). Zhang et al. investigated the impact of the PD-1 pathway on macrophage polarization toward the M2 phenotype (145). Their findings revealed that a PD-1 agonist (PD-L1 Fc) induced macrophage polarization toward the M2 phenotype and enhanced their phagocytic activity. Additionally, the authors provided indirect evidence that PD-1 signalling could modulate macrophage polarization through metabolic reprogramming, potentially regulated by the PI3K/AKT/mTOR and MEK/ERK signalling pathways (145). However, this conclusion was obtained in a study of human pregnancy. Further studies are required to confirm whether the potential mechanisms identified are also applicable to tumours. The current evidence does not provide a thorough or direct explanation of the mechanisms underlying the impact of the PD-1/PD-L1 signalling pathway on M2 TAM polarization. Therefore, there is a need for future research to comprehensively clarify this issue.

### 7.6 CTLA4

CTLA-4 (cluster of differentiation 152, CD152) is a receptor expressed on the surface of activated T cells. CTLA-4 expression is usually seen in T-cell activation, but Tregs constitutively express CTLA-4 due to their high levels of the forkhead transcription factor FoxP3. CTLA-4 functions mainly by competing with the CD28 receptor to bind B7 ligands (B7-1/CD80 and B7-2/CD86) on antigen-presenting cells (APCs) (146). In the TME, the CTLA-4 receptor binds with higher affinity and lower density to the B7 ligand on the surface of macrophages, thereby outperforming the CD28 receptor on T cells with regard to binding to the B7 ligand and blocking the second activation signal necessary for T cells. Professor Allison demonstrated the negative effects of CTLA-4, demonstrating binding of CTLA-4 to the B7 ligand. The research showed that CTLA-4 binding to the B7 ligand inhibits T-cell secretion of IL-2 and T-cell proliferation following TCR activation and that blocking CTLA-4 with anti-CTLA-4 antibodies leads to tumour rejection. In addition, the Fc fragment of the anti-CTLA-4 antibody interacts with IgG Fc (FcγR, antibody-coated by FcγR-expressing cells) through ADCC process clearance and/or antibody-dependent cell-mediated phagocytosis (ADCP) of specific host cell receptors. Multiple laboratories have reported that interaction of CTLA-4 antibodies with activated Fc receptors on host cells is critical for selective depletion of Tregs in tumours, leading to tumour rejection (146). Yofe et al. examined the effects of anti-CTLA-4-blocking, Tregcell-depleting and FcR-engaging activity on the immune response within tumours by using single-cell RNA sequencing. Their findings indicated that immune remodelling was not solely driven by Tregcell depletion or CTLA-4 blockade, but primarily through FcγR engagement, downstream activation of type I interferon signalling, and reduction of suppressive macrophages (147). Current research has primarily concentrated on the interaction between CTLA-4 antibodies and FcγR. However, the impact of CTLA-4 on macrophage polarization remains unclear and necessitates further investigation. Cytotoxic T lymphocyte-associated antigen 4 (CTLA4) shows potent antitumour effects, ipilimumab has been approved by the FDA for treatment of patients with advanced melanoma, and tremelimumab is still being investigated in clinical trials (148). Despite positive clinical outcomes, in a recent phase III trial, 28% of melanoma patients receiving ipilimumab (anti-CTLA4) experienced grade 3 and 4 immune-related adverse reactions (irAEs), manifesting as multiorgan toxicity (149).

Clinically tested anti-CTLA-4 antibodies have been shown to have less efficacy and highly toxicity, and Liu and colleagues suggest that the CTLA-4 checkpoint should be preserved rather than inhibited (150). Compared to anti-PD therapy, CTLA-4 targeted therapy has two related challenges: poor efficacy and increased toxicity. With monotherapy, durable response rates are significantly high, and safety is manageable; however, more than 50% of patients do not respond to this treatment. The CheckMate-032 study evaluated the safety and efficacy of nivolumab in combination with ipilimumab in patients with chemotherapy-resistant oesophago-GC in Western countries. The study showed that nivolumab in combination with ipilimumab had clinically meaningful antitumour activity, a durable response, led to an encouraging long-term OS, and showed a manageable safety profile (151). An investigator-initiated, single-arm, open-label, 14-centre phase 2 trial of nivolumab in combination with low-dose eprilimus for first-line treatment of MSI-H GC, the NO LIMIT study (WJOG13320G/CA209-7 W7), is ongoing; 28 patients with unresectable advanced, recurrent or metastatic gastric or combined oesophago-GC and histologically confirmed adenocarcinoma were enrolled, with the primary objective of determining the overall response rate (ORR) of the NIVO + IPI regimen through a blinded, independent centre review (128). Although nivolumab combined with ipilimumab did not improve progression-free survival (PFS) or the objective remission rate (ORR) compared with chemotherapy in PD-L1 CPS ≥ 5 or all randomised patients in the study by Shitara et al, treatment with combined nivolumab and ipilimumab showed clinically meaningful antitumour activity and a manageable safety profile in severely pretreated patients with advanced gastroesophageal cancer (152).

Immunotherapy is still an emerging strategy in cancer treatment. Most therapeutic studies of immune checkpoints in GC have been limited to animal models, and evaluation of clinical treatment strategies is lacking. A number of clinical trials for immune screening are underway (more details are shown in Tables file. **Table 2**.).

**Table 2:** Selected ongoing clinical trials exploring of immune checkpoint inhibitors (ICIs) in gastric cancer.

Traget	Agent	Conditions	Arms	Phase	NCT number	Recruitment Status
**PD-1**	Nivolumab	Recurrent/Metastat-ic Gastric Cancer	Nivolumab Paclitaxel	1,2	NCT05535569	Completed
**PD-1**	Nivolumab	Gastric Cancer	Nivolumab plus SOX Nivolumab	2	NCT04782791	Not yet recruiting
**PD-1**	Nivolumab	Esophagus Cancer Adenocarcinoma Stomach Cancer	Rucaparib Ramucirumab Nivolumab	1,2	NCT03995017	Recruiting
**PD-1**	Tislelizumab	Advanced Gastric Cancer Advanced Gastroesophageal Junction Adenocarcinoma	Tislelizumab SOX(S-1+ Oxaliplatin)	2	NCT04890392	Recruiting
**PD-1**	Tislelizumab	Advanced Gastric Cancer	Transcatheter Arterial Chemoembolizat-ion	2	NCT04799548	Recruiting
**PD-1**	Pembrolizumab	Gastric and Gastroesophageal Junction Adenocarcinoma	Pembrolizumab Trifluridine/Tipir-acil	1,2	NCT05508737	Not yet recruiting
**PD-1**	Pembrolizumab	Oesophageal Squamous Cell Carcinoma Gastric Cancer Hepatocellular CarcinomaColorec-tal Cancer Oseophageal Adenocarcinoma	Pembrolizumab THOR707 Cetuximab	2	NCT05104567	Active, not recruiting
**PD-1**	Pembrolizumab	Gastric Cancer	Pembrolizumab Trastuzumab Capecitabine Cisplatin	1,2	NCT02901301	Active, not recruiting
**PD-1**	Pembrolizumab	Gastric Cancer Gastroesophageal Junction Cancer	Pembrolizumab Placebo Cisplatin Capecitabine 5-fluorouracil Docetaxel Oxaliplatin Leucovorin	3	NCT04882241	Recruiting
**PD-1**	Pembrolizumab	Gastric Adenocarcinoma Gastroesophageal Junction Adenocarcinoma	Pembrolizumab paclitaxel	3	NCT02370498	Completed
**PD-1**	Sintilimab	Gastric Cancer	Sintilimab SOX (oxaliplatin+Teg-afur)	2	NCT05594381	Not yet recruiting
**PD-L1**	Avelumab	Gastroesophageal Junction Adenocarcinoma Stomach Neoplasms	Avelumab addition to perioperative chemotherapy	2	NCT03979131	Recruiting
**PD-L1**	Avelumab	Unresectable, Locally Advanced or Metastatic, Adenocarcinoma of the Stomach, or of the Gastro Esopha-geal Junction	Avelumab Oxaliplatin 5-Fluorouracil Leucovorin Capecitabine	3	NCT02625610	Completed
**CTLA-4**	Ipilimumab	Lung Cancer Liver Cancer Colorectal Cancer Pancreas Cancer Ovary Cancer Cervical Cancer Head and Neck Cancer Breast Cancer Gastric Cancer Esophageal Cancer Sarcoma	ipilimumab pembrolizumab durvalumab	1,2	NCT05187338	Recruiting
**CTLA-4**	Ipilimumab	Gastric Cancer	OTSGCA24 Nivolumab Ipilimumab	1	NCT03784040	Recruiting

(fromhttp://clinicaltrials.gov).

### 7.7 CAR-M

Many current cancer treatment strategies, including use of ICIs and cancer vaccines, aim to enhance the natural immune capacity of adaptive immune cells, such as cytotoxic T lymphocytes (CTLs), to recognize cancer neoantigens. However, not all tumours exhibit an antigenic component that activates the immune system, and for such tumours, transfer of T cells with chimeric antigen receptors (CARS) or of genetically engineered T cells is a new therapeutic strategy (153). Relay cell therapy using chimeric antigen receptor (CAR) immunotherapy is currently making great progress in haematologic malignancies, but its use in solid tumours has been a challenge. Some of the major barriers to CAR immunotherapy for solid tumours include the manufacture of CAR T cells, lack of tumour-specific antigens, inefficient translocation and infiltration of CAR T cells to the tumour site, immunosuppression of the TME, treatment-related toxicity and antigen escape (154). The unique effector function and ability of macrophages to penetrate tumours may overcome current barriers in CAR-T-cell treatment of solid tumours, and macrophages more readily localise and persist in the TME (155). Current research in designing CAR-M has found that the basic CAR design principles of the T-cell field apply to macrophages. Conventional CARS are modular transmembrane proteins consisting of an extracellular antigen recognition structural domain and one or more cytoplasmic signalling domains (156).

Macrophages can be precisely modified through use of modern techniques, such as viral vector design or genome editing. Klichinsky and colleagues used a chimeric adenoviral vector (Ad5f35) that efficiently mediates gene transfer into human macrophages, resulting in high and sustained expression of CAR (106). Anti-HER2 CARs were transformed into Ad5f35 vectors and transduced with primary cultured human peripheral blood monocyte-derived macrophages. These anti-HER2 CAR-MS effectively induced phagocytosis in the HER2+ ovarian cancer cell line SKOV3. Mice injected with the HER2+ ovarian cancer cell line SKOV3 treated with a single dose of HER2-targeted CARMs (intravenous or intraperitoneal) showed a significant reduction in tumour load and prolonged survival, but the tumours of all mice eventually progressed (157). Notably, the evidence presented in this study suggests that CAR-M transduced with the Ad5F35 vector secretes a range of proinflammatory cytokines, inducing a proinflammatory M1 phenotype (157). Furthermore, *in vitro* coculture experiments have demonstrated that CAR-Ms induces a proinflammatory phenotype in M2-type macrophages, activation and maturation of dendritic cells, and recruitment of resting and activated T cells. HER2-targeted CAR-Ms and donor-derived polyclonal T cells lead to a better antitumour response in mice treated with metastatic SKOV3 transplants than in mice treated with CARM or T cells alone. This synergistic effect may be due to enhanced antitumour activity of T cells by CAR-M (157). In another study, Morrissey et al. designed a new CAR structure, the chimeric antigen phagocytic receptor (CAR-P), using the unique phagocytic properties of macrophages (158). The design of CAR-P is similar to that of the classical anti-CD19 CAR used for T cells, but it screens for various mouse phagocytic receptors, such as FcRγ, BAL1 and MerTK, and uses them as intracellular signalling domains for CARS. This study demonstrates that different CAR-P designs can promote phagocytosis of various cancer-associated antigens (158). Niu et al. similarly achieved tumour killing by CAR-M using CCR7-targeted CAR-M in the RAW264.7 cell line (159). These CAR-M cells exhibited antigen-specific cytotoxic effects *in vitro* and led to prolong survival and prevent metastasis to distant tissues survival in a 4T1 breast cancer model. Subsequent macrophage treatment also increased serum levels of the proinflammatory cytokines IL1-b, IL-6 and TNF-α (159). Another CAR-MS was designed to target HER2-expressing cancers while activating the CD147 signalling domain to induce matrix metalloproteinases to destroy the TEM but caused no changes in phagocytosis, killing or cytokinin release. In the HER2 + 4T1 breast cancer model, CAR-M slowed tumour growth by reducing its collagen content, enhancing the presence of T cells and increasing IL-12 and interferon-g signalling, directly targeting the tumour ECM rather than tumour cells (160). Recently, Zhang et al. investigated generation of efficient CAR-Ms from iPSCs, which were able to reduce tumour growth and activate phagocytosis in tests against leukaemia, ovarian and pancreatic cancer cell lines (161).

At present, use of CAR macrophages is still immature, with only one phase I clinical trial initiated for CAR macrophages (NCT04660929) and successful preparation of CT-0508 (anti-HER2-CAR-M), which has been approved by the Food and Drug Administration (FDA) for clinical studies to test CT-0508 in HER2-positive adenocarcinomas, though no results have been reported. The Centre Oscar Lambret in Lille, France, is currently conducting a prospective study (CARMA-2101) to investigate the antitumor activity of novel CAR-Ms using organoids derived from 100 breast cancer patients. This study specifically aims to evaluate the impact of CAR-Ms on organoids derived from HER2-negative, HER2 low, and HER2-positive breast cancer. The study will also compare the activity of CAR-Ms with non-modified macrophages (NCT05007379). Although, many limitations are not yet apparent. Taken together, these pioneering studies demonstrate the ability of CAR-M to infiltrate the tumour ecological niche and initiate a broad antitumour response through the host immune system, demonstrating the great potential of CAR-Ms to provide new ideas for immunotherapy with macrophages in GC.

## 8 Conclusion

GC is the fifth most common cancer worldwide and the third leading cause of cancer death (1). Combination therapy based on surgical treatment is currently the mainstay of GC treatment. Despite the therapeutic efficacy of combination therapy, chemoresistance still leads to poor prognosis. The TME is involved in suppression of chemoresistance and the antitumour immune response in GC (5). Macrophages are the most abundant inflammatory cells in the TME, and there is crosstalk between macrophages and the TIME, with the M1 phenotype acting as a proinflammatory agent and the M2 phenotype promoting tumour progression (7). Macrophages in GC interact with immune cells, tumour cells, vascular endothelial cells and flora (especially *H. pylori*) to promote cancer progression and lead to treatment resistance. In recent years, targeting macrophages has offered new hope for immunotherapy for GC. By reducing infiltration of TAMs, reverting the M2 phenotype to the M1 phenotype and depleting M2-polarised TAMs, patients can be effectively treated of malignant disease. The discovery of immune checkpoints such as CTLA-4 and PD-1/PD-L1 also offers new hope for GC patients (135), and ICIs offer better efficacy and lower toxicity than chemotherapeutic agents. CAR-M is emerging as a strategy in immunotherapy for tumours that do not exhibit antigenic components that activate the immune system (154). In clinical practice, combination of chemotherapy and immunosuppressive therapy is beneficial for patients with advanced GC.

## Author contributions

JZ: Writing – original draft. CH: Writing – review & editing, Writing – original draft. RZ: Writing – original draft. JX: Writing – original draft. YZ: Writing – review & editing. LY: Writing – review & editing. SZ: Writing – review & editing. SP: Writing – review & editing. MC: Writing – review & editing. JQ: Data curation, Writing – review & editing. XC: Funding acquisition, Writing – review & editing. ZX: Writing – review & editing.

## Glossary

**Table:**

5-FU	5- fluorouracil
ADCC	antibody-dependent cytotoxicity
AP-1	activator protein-1
APCs	antigen-presenting cells
bFGF	basic fibroblast growth factor
BM	basement membrane
CagA	Cytotoxin-associated gene A
CAR	chimeric antigen receptor
CAR-P	chimeric antigen phagocytic receptor
CCL	chemokine ligand.
CCLR/CCR2	chemokine ligand receptors/Chemokine receptors 2
CDC	complement-dependent cytotoxicity
CIITA	class II major histocompatibility complex transactivator
COX-2	cyclo-oxygen-ase-2
CRC	colorectal cancer
CSF-1/CSF-1R	colony Stimulating Factor -1/colony Stimulating Factor receptor 1
CSF-1	colony stimulating factor 1
CTLA-4	cytotoxic T lymphocyte-associated protein 4
CTLs	cytotoxic lymphocytes
CXCL	chemokine (C-X-C motif) ligand
DCs	dendritic cells
DDP	cisplatin
ECM	extracellular matrix
EGF	epithelial growth factor
EGFR	epidermal growth factor receptor
EMT	epithelial-mesenchymal transition
FDA	Food and Drug Administration
FLT1	Fms-associated tyrosine kinase 1
*Fn*	Fusobacterium nucleatum
GC	gastric cancer
gCM-MNS	genetically engineered cell membrane-encapsulated magnetic nanoparticles
GC‒MSCs	GC-derived mesenchymal stem cells
GIT	gastrointestinal tumours
GM-CSF	granulocyte-macrophage colony stimulating factor
GPR43	G protein-coupled receptor 43
*H. pylori*	Helicobacter pylori
H2O2	hydrogen peroxide
HDACi	histone deacetylase inhibition
HGF	H hepatocyte growth factor
HIF-1α	hypoxia inducible factor-1α
HLA	human leukocyte antigen
ICIs	immune checkpoint inhibitors
IECs	intestinal epithelial cells
IFN-γ	interferon-γ
IL	interleukin
iNOS	nitric oxide synthase
irAEs	immune-related adverse reactions
IRAK	interleukin receptor associated kinase
IRF	interferon regulatory factor
JMJD3	J histone demethylase Jumonji D3
KLF	Krüppel-like Factor
lncRNA	long-stranded noncoding RNA
LPS	lipopolysaccharide
M2-exo miR-21	M2 macrophage-derived exosome miR-21
MABs	monoclonal antibodies
MAP	multiple antigen peptide
MDSCs	M myeloid suppressor cells
MLMs	monocyte-like macrophages
M-MDSC	monocytic myeloid suppressor cell
MMPs	Matrix metalloproteinases
MVD	microvascular density
NF-kB	nuclear factor-k-gene binding
NK	natural killer cells
NLRs	NOD-like receptors
NOCs	N-nitroso compounds
NOD1	nucleotide-binding oligomerization domain-containing protein 1
NOVA1	neuro-oncological ventral antigen 1
O2-	superoxide anions
OH-	O hydroxyl radicals
ORR	objective remission rate
ORR	overall response rate
*P. acnes*	Propionibacterium acnes
PAMPs	pathogen-associated molecular patterns
PD-1/PDL-1	programmed death 1/programmed death ligand 1
PDGF	platelet-derived growth factor
PFS	progression-free survival
PI3K	The phosphoinositide 3-kinase
PlGF	placental growth factor
PPARγ	peroxisome proliferator-activated receptor γ
PPARδ	peroxisome proliferator-activated receptor δ
PRRs	pattern recognition receptors
ROS	reactive oxygen species
SDA	State Drug Administration
SIRPα	signal-regulated protein alpha
STAT	S transcription
TAMs	tumour-associated macrophages
TGF-β	transforming growth factor-β
Th cell	helper T cell
TIME	tumour immune microenvironment
TLRs	Toll-like receptors
TME	tumour microenvironment
TNF-α	tumour necrosis factor-α
Tregs	regulatory T cells
VacA	vacuolar toxin A
VEGF	vascular endothelial growth factor
YAP1	Yes-associated Protein1
